# Active Semi-Supervised Community Detection Based on Must-Link and Cannot-Link Constraints

**DOI:** 10.1371/journal.pone.0110088

**Published:** 2014-10-17

**Authors:** Jianjun Cheng, Mingwei Leng, Longjie Li, Hanhai Zhou, Xiaoyun Chen

**Affiliations:** School of Information Science and Engineering, Lanzhou University, Lanzhou, Gansu Province, China; Xiamen University, China

## Abstract

Community structure detection is of great importance because it can help in discovering the relationship between the function and the topology structure of a network. Many community detection algorithms have been proposed, but how to incorporate the prior knowledge in the detection process remains a challenging problem. In this paper, we propose a semi-supervised community detection algorithm, which makes full utilization of the *must-link* and *cannot-link* constraints to guide the process of community detection and thereby extracts high-quality community structures from networks. To acquire the high-quality *must-link* and *cannot-link* constraints, we also propose a semi-supervised component generation algorithm based on active learning, which actively selects nodes with maximum utility for the proposed semi-supervised community detection algorithm step by step, and then generates the *must-link* and *cannot-link* constraints by accessing a noiseless oracle. Extensive experiments were carried out, and the experimental results show that the introduction of active learning into the problem of community detection makes a success. Our proposed method can extract high-quality community structures from networks, and significantly outperforms other comparison methods.

## Introduction

Community structures are significant features observed in many complex networks, meaning that the nodes in a network can be divided naturally into groups, within which connections are relatively dense but between which connections are much sparser. Communities may correspond to the sets of topic-related Web pages in Web graphs [1]–[3], the papers on certain scientific research subjects in article citation networks [4], [5], the real social groupings in social networks [6]–[9], or the basic reaction modules or other functional units in metabolic networks and protein-protein interaction networks [3], [10]–[15]. Thus, community structure detection is of great importance because it can shed light on the relationships between the structural and functional characteristics of networks. Furthermore, a number of research results have provided evidence that networks may have quite different properties when considered from a community perspective rather than from the perspective of individual nodes or a whole network [3], [11], and therefore, many interesting network features may be revealed through detecting the community structures from networks.

Community detection has therefore attracted significant interests from researchers, and a large number of community detection methods and algorithms have been developed during the last decade. For example, the GN algorithm [6], [7] is a divisive community detection algorithm, the Fast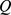 algorithm [8] and the CNM algorithm [16] are agglomerative algorithms. Also, Fast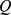 and CNM are modularity-optimization based algorithms, which take the modularity [7] as the optimization objective, and try to maximize the modularity over all possible community structures of a network. In addition to these, all of the methods in [10]–[12], [17]–[19] are based on the modularity-maximization strategies. Spectral methods based on the eigenvalue spectra of various types of matrices associated with networks have also yielded fruitful results [10], [11], [20]–[26] in discovering community structures from networks. The LPA algorithm [27] exploits a label propagation mechanism to make the densely connected groups of nodes to reach consensuses on node labels to form communities, and a series of variants and improvements [28]–[30] have been derived from LPA owing to its simplicity and near linear-time complexity. Methods based on random walk utilize the tendency of a random walker to identify community structures from networks, the walker tends to be trapped in communities rather than walks across community boundaries within a limited number of steps. Such methods have also been applied in many applications successfully [31]–[38]. In which, the Infohiermap (abbreviation for Hierarchical Infomap [36]) algorithm [37], which reveals the best hierarchical community structures in networks by finding the shortest multilevel descriptions of the random walker, and the PPC (acronym for Personalized PageRank Clustering) algorithm [38], which combines the random walks and the modularity to efficiently identify the community structures of networks, are two representatives of the state-of-the-art algorithms based on random walk.

All of these algorithms and methods are in essence a kind of unsupervised learning, meaning that they identify community structures from networks using only topological information of the networks, without using any prior knowledge of the nodes. However, in many real-world applications, there exists usually some background information, which can be used as the guidance in detecting the communities from networks. The *must-link* and *cannot-link* constraints are one type of such background information, which are also known as the pair-wise constraints that specify whether the nodes involved must or cannot be classified into the same communities. If the relationship between two nodes is “*must-link*”, the two nodes must be assigned to the same community. If the relationship is “*cannot-link*”, the two nodes cannot be classified into the same community, and they must be allocated into different communities. The *must-link* and *cannot-link* constraints are generally adopted as a type of semi-supervised information and have been successfully integrated in many clustering algorithms to improve their performance. To some extent, the essence of community detection is node clustering in networks. Therefore, it is a natural idea to introduce these constraints to guide the process of community detection. However, this remains a challenge, and the first problem to be addressed is how to obtain the high-quality semi-supervised components. In general, the semi-supervised components are acquired by annotating the data points involved by an oracle (e.g. a domain expert). In order to maximize the utilities of the semi-supervised components at the minimum cost, strategies based on active learning [39] are used to actively select those data points to annotate, such that the clustering algorithm can achieve as a high performance as possible compared with random selection. Most active learning algorithms are pool-based [40], [41] or stream-based [41]–[43], and most work with data represented by attribute vectors [44]–[46]. However, for the problem of community detection, the nodes in the networks have no other attributes except for the topological information, thus these algorithms cannot be utilized directly. As datasets with intrinsic graph structures become ubiquitous, substantial efforts have been devoted in recent years to the problem of active learning on graphs, and many algorithms [47]–[50] have been proposed.

The main contributions of this paper are threefold. First, we propose a semi-supervised community detection algorithm, which fully utilizes the *must-link* and *cannot-link* constraints to guide the procedure of community detection to extract high-quality community structures from networks. Next, for being used in the proposed semi-supervised community detection algorithm, we propose an algorithm based on active learning to actively select the nodes with maximum utilities from the networks to generate the *must-link* and *cannot-link* constraints. This active learning algorithm takes both the informative nodes and the nodes with least certainty into account, and thus it can select the nodes with local maximal degrees and the nodes located at the boundaries between the ground truth communities step by step to access a noiseless oracle for generating the *must-link* and *cannot-link* constraints. Finally, we carried out extensive experiments on several real-world networks to evaluate the performance of our proposed method, the experimental results demonstrate that the method can extract high-quality community structures from networks, and outperforms other comparison methods significantly.

## Definitions

To facilitate the description of our algorithms, the following notations are given in definition form:

**Definition 1** *A network is a graph* 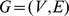 *, where* 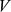 *and* 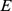 *are the node set and the edge set, respectively, and* 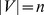, 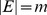.

In this paper, we only consider the simple networks as what are involved in the conventional problem of community detection, which means that all of the networks involved are undirected and unweighted graphs, and every edge must connect two different nodes.

**Definition 2** *The community structure of a network is a partition* 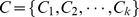 *of the network, subject to the conditions* 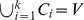 *and* 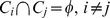 *, where* 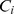 *represents the node set of community* 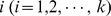 *, and* 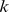 *is the number of communities.*

Compared with the general concept of a partition in graph theory, another condition, 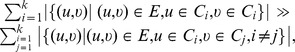 must be attached to the *community structure*, which indicates that the connections between intra-community nodes are much denser than those between inter-community nodes.

**Definition 3** *The must-link constraint set,* 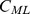 *:* 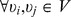 *,* 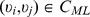 *indicates that two nodes* 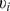 *and* 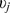 *must belong to the same community.*

**Definition 4** *The cannot-link constraint set,* 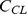 *:* 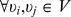 *,* 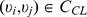 *indicates that two nodes* 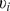 *and* 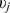 *cannot be classified into the same community, and they must be allocated into different communities.*

As only undirected and unweighted networks are considered in this paper, the tuples in 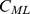 and 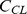 are order-independent, i.e., 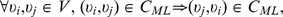 and 
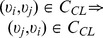
 also.

**Definition 5** 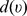 *is the degree of node* 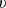 *, that is, the number of edges associated with node* 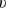 *.*

**Definition 6** *For a given node* 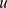 *,* 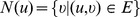 *is a set containing all neighbors of node* 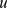 *.*

**Definition 7** 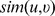 *is the similarity measure between two nodes,* 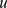 *and* 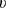 *.*

**Definition 8** *The similarity measure between community* 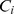 *and node* 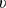 *, denoted as* 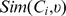 *, is formulated as follows:*

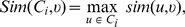
which means that it is defined as the maximal value of similarity between every node in community 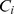 and node 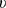.

## Methods

### Semi-supervised community detection algorithm

As mentioned above, the proposal is a semi-supervised algorithm, which makes full utilization of the *must-link* and *cannot-link* constraints to guide the process of community detection. The pseudo-code outlining the procedure of our algorithm is shown as Algorithm 1 in Table 1.

**Table 1:** Algorithm 1: Semi-supervised community detection algorithm based on *must-link* and *cannot-link* constraints.

** Input:** 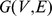 , the network; 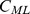 , the *must-link* constraint set; 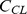 , the *cannot-link* constraint set
** Output:** 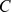 , a partition of the network corresponding to the community structure
1: Augment the *must-link* and *cannot-link* sets utilizing the transitive property of *must-link*:
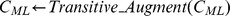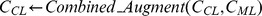/* *Construct the initial skeleton of the community structure from the cannot-link and must-link constraints* */
2: Initialize set 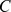 corresponding to the community structure, and set 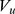 used to record the unclassified nodes:
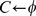
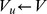
3: Take every node involved in each *cannot-link* constraint 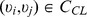 as a separate community:
**foreach** 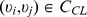 **do**
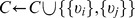
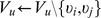
4: If some nodes contained in different communities 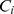 , 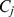 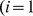 , 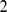 , 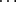 , 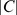 , 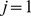 , 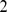 , 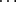 , 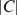 , 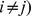 are involved in some *must-link* constraints, then merge community 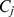 into community 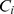 :
** for** 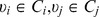 **do**
** if** 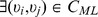 **then**
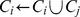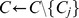/* *Expand the communities to obtain the final community structure* */
5: For each community 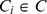 , select those unclassified nodes that have *must-link* and transitive *must-link* relationships with the nodes contained in 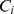 , and insert them into 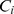 , repeatedly:
** foreach** 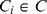 **do**
** repeat**
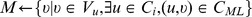
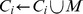
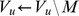
** until** 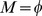
6: Among all communities and unclassified nodes, find the most similar pair 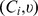 from the network greedily globally, and insert the node 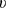 into the community 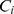 first:
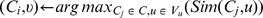
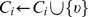
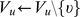
and then insert the nodes that have *must-link* and transitive *must-link* relationships with node 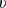 into community 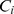 :
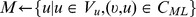
** repeat**
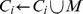
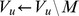
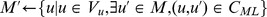
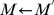
** until** 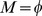
7: Repeat step 6, until all nodes in the network are processed, i.e., until 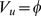
8: **return** 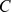

A set of *must-link* constraints define a transitive relation over the nodes involved, and permit additional *must-link* constraints to be derived from the original set, e.g., 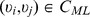, and 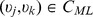 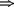 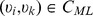. The *cannot-link* constraints themselves do not have the transitive property, but the combination of *cannot-link* constraints and *must-link* constraints also permits additional *cannot-link* constraints to be inferred, e.g., 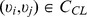 and 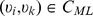 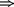 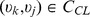.

Thus, in Algorithm 1, we start with the derivations of *must-link* constraints, and enlarge set 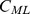 by adding all derived constraints, which are the functions of 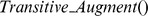. Then, from the combination of *cannot-link* constraints and enlarged *must-link* constraints, all additional *cannot-link* constraints are inferred, and set 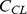 is augmented by adding all of the inferences, which is conducted using the 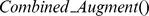 function.

For any node pair in the *cannot-link* constraints, the two nodes involved must be classified into different communities, and we therefore use the *cannot-link* constraints to construct the initial skeleton of the community structure. For any *cannot-link* constraint node pair, we create two new communities and insert the two nodes into each of the communities, respectively, i.e., for any node pair 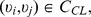 two communities 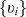, 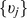 are created. In this way, we obtain many communities with a sole member in each of them. Among all of the communities, two members across two communities may have a *must-link* relationship, e.g., for node 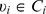 and node 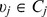, 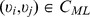 may exist. According to the definition of the *must-link* constraints, nodes 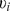 and 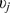 should be in the same community. Therefore, we merge the two communities involved into one — in Algorithm 1, community 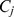 is merged into community 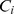 using the operation 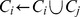. After all cases of this type are processed, we obtain the initial skeleton of the community structure, and those nodes in the initial communities are intended to be seeds or initiators of the corresponding communities.

Then, based on the skeleton of the community structure, we begin to expand the communities. First, if some unclassified nodes (nodes that have not been allocated to any community yet) have *must-link* or transitive *must-link* relationships with some classified nodes (nodes that have already been assigned to communities), the unclassified nodes are allured into joining the communities in which their buddies belong. Concretely, for every community 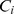 and any node 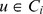, the algorithm selects the unclassified nodes that have *must-link* and transitive *must-link* relationships with 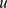, and inserts them into community 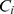.

After all of this type of *must-link* node pairs are processed, a greedy strategy is employed in the next steps: the (*community, unclassified node*) pair 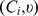 with the largest value of similarity between the community and unclassified node is chosen from all (*community, unclassified node*) pairs, and the algorithm inserts node 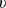 into the corresponding community 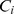, which means that in each iteration, a global optimal node is selected and assigned to the corresponding community. In the next steps, we find all nodes that have *must-link* and transitive *must-link* relationships with node 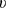, and insert them into community 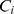 as well. These greedy operations are repeated until every node in the network is classified into the corresponding community, and we finally obtain the resulting community structure.

### Active learning algorithm

In this subsection, we present the idea of the proposed semi-supervised component generation algorithm based on active learning. Generally, the semi-supervised components are obtained by annotating the nodes involved by a noiseless oracle. However, in real-world applications, annotating the nodes in networks is a time-consuming job, and it is also very costly. Therefore, the goal of the proposed algorithm is to select those nodes with the maximum utilities for Algorithm 1 to generate the semi-supervised components.

In Algorithm 1, the initial skeleton of the community structure is constructed purely from the *must-link* and *cannot-link* constraints. The nodes involved in the constraint pairs are taken as the seeds or initiators of the communities, and the communities are then expanded by pulling the most similar nodes to join the corresponding community iteratively. From this perspective, the selected nodes should cover all of the ground truth communities and have relative larger degrees, such that the accuracy of community assignments of the nodes can be ensured during the expansion process. However, most of the nodes having a larger degree are the internal nodes of the ground truth communities, and are unlikely to be assigned to a wrong community. The nodes located at the community boundaries tend to be misclassified, but their selection does not facilitate the expansion of the communities. We make a compromise to select those nodes with a relative larger degree and the boundary nodes to generate the *must-link* constraints and the *cannot-link* constraints by accessing the oracle. The basic idea of this active learning algorithm is to extract some nodes with larger degrees in local area into a set and partition the set into some clusters quickly, then to select the nodes having the maximal degree values in each cluster and the nodes having connections with other nodes in other clusters to access the oracle to query the relationship between some pairs of the selected nodes. Which means we try to maximize the utilities of the semi-supervised components by taking both the informative nodes (the nodes with a relative larger degree) and the nodes with least certainty (the boundary nodes) into account during the process of node selection.

**Figure 1:**
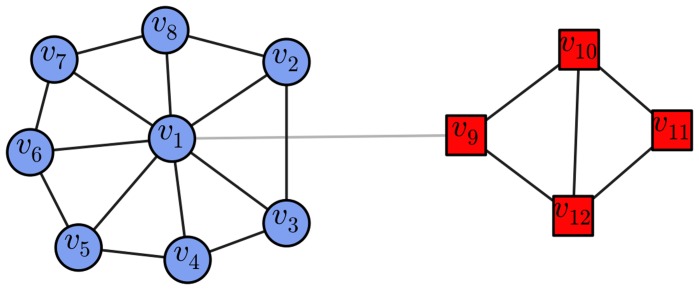
A simple two-community network. If the nodes are selected according to their degree values, only node 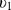 will be selected, and community 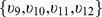 will be ignored. However, using the *score* value in conjunction with degree value of every node in the network as the condition, we will select node 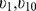 (or 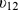) from the network at least, which means that the selected nodes can cover all of the ground truth communities. (The different node shapes and shades indicate different communities, the black lines are the edges within communities, and the light-gray connections represent the edges across different communities. This illustration style is also applied in the following figures.)

Although Algorithm 1 needs nodes with larger degrees to be taken as community seeds to facilitate the expansion of the communities, if we select nodes using only their degrees as a condition, the nodes in small communities will necessarily be ignored. For example, in the simple two-community network illustrated in Figure 1, only node 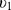 will be selected according to the values of the node degrees. It is obviously that the selected nodes do not cover all of the ground truth communities. To solve this problem, we calculate a degree-related *score* for every node 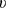 in the network using the following formula:
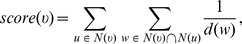



and the *score* values of nodes are used in conjunction with the degree values of nodes as a condition for node selection.

Concretely, the nodes whose *score* values are larger than a given threshold, 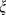, are extracted into a set, *cand*, as candidates firstly, and *cand* is then partitioned into some clusters by calling the function 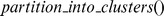. From every cluster, the node with the maximal degree is selected as the representative of that cluster, and the ties are broken by selecting the node with both the maximal degree and the maximal *score* value. In this way, at least nodes 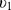 and 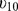 (or 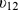) will be selected from the network illustrated in Figure 1 after these steps. Using these operations coupled with the following steps, we can ensure that the selected nodes distribute over all of the ground truth communities.

For the selected representatives, we access the oracle to query the relationship between each pair of them, and generate *must-link* constraints or *cannot-link* constraints according to the query results. There may exist other nodes having the same maximal degree with the representative in each cluster, thus we process these nodes in descending order of the degree values of the cluster representatives. From each cluster, we draw out every one of such nodes and access the oracle to query the relationship between the node and the representative of that cluster. If the query result indicates that the relationship is “*cannot-link*”, then the relationships between the node and other representatives are queried. If some of the results show that the node and certain representatives have *must-link* relationships, we insert the node into the corresponding clusters. And if all the results are “*cannot-link*”s, then a new cluster is constructed by taking the node as its representative. During this process, the *must-link* constraint set or the *cannot-link* constraint set is updated according to the result of each query. This process is repeated until all nodes having the same maximal degrees with the representative nodes are processed, or until certain user-specified termination criteria, such as the query number limit, *etc.*, are reached. The initial *must-link* and *cannot-link* constraints are then obtained, and the nodes with the maximal degrees in all clusters will cover all of the ground truth communities.

If more constraints are needed, the boundary nodes of the clusters are considered in order of the numbers of nodes contained in the clusters alternately, where the boundary nodes are those having edges connected with nodes located in other clusters. From each cluster, the boundary node with the maximal degree is selected, and the algorithm accesses the oracle to query the relationship between the boundary node and the representative of that cluster. If the relationship is “*cannot-link*”, then the relationships between the boundary node and other representatives are queried. If some of the results show the *must-link* relationships between the boundary node and certain representatives, we insert the boundary node into the corresponding clusters. As with the process for nodes with the maximal degrees, during the process for each boundary node, the *must-link* constraint set or the *cannot-link* constraint set is updated after each query. This process is repeated until all boundary nodes are selected, or certain user-specified termination criteria are met. Finally, all *must-link* and *cannot-link* constraints generated are returned and utilized in Algorithm 1.

The steps of the entire procedure are listed as Algorithm 2 in Table 2.

**Table 2:** Algorithm 2: Active approach to generate the *must-link* and *cannot-link* constraints.

** Input: ** 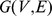 , the network; 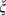 , the 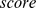 threshold
** Output: ** 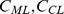 , the sets of *must-link* constraints and *cannot-link* constraints
1: For each node 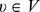 , calculate a 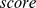 :
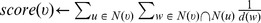
2: Extract the nodes whose 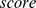 values are larger than the given threshold, 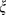 , from 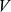 into set 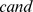 as candidates
3: 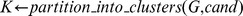
4: Select the node with the maximal degree in each cluster 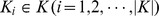 as its representative
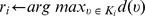
if more than one node having the same maximal degree exist, the node with the maximal 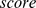 value is chosen
5: Initialize the sets of *must-link* constraints and *cannot-link* constraints:
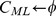 ; 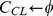
6: For any two representatives 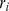 , 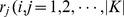 , and 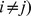 , access the oracle to query their relationship:
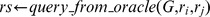
if 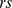 = “*must-link*” then 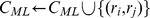
if 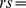 “*cannot-link*” then 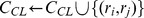
7: Check each cluster 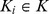 in descending order of the degree values of the representative nodes, and select each node 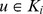 that has the same maximal degree with 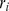 to query the relationships between 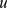 and 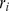 :
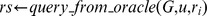
• if 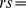 “*must-link*” then update the *must-link* set: 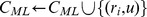
• if 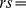 “*cannot-link*” then update the *cannot-link* set and 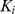 first:
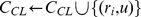 ; 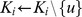
then query the relationships between *u* and other representatives 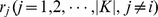 :
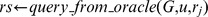
• if the query result is “*must-link*”, update 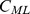 and 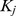 : 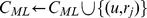 ; 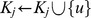
• if the result is “*cannot-link*”, update 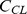 : 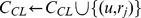
• for all 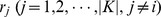 , if all query results are “*cannot-link*”s, create a new cluster by taking 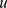 as its representative:
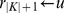 ; 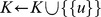
8: Repeat step 7, until all nodes having the same maximal degrees in every cluster are processed, or until certain user-specified termination criteria are met
9: If more *must-link* constraints and *cannot-link* constraints are needed, consider the boundary nodes in each cluster 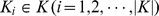 alternately in order of the number of nodes contained in 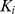
• From the remainder nodes in 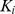 , extract the boundary nodes into set 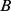 , where the boundary nodes are those nodes having edges connected with nodes in other clusters
• if 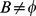 , select the node with the maximal degree, denoted as 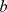 , from 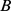 to query the relationship between 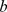 and the representative of 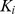 :
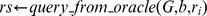
• if 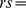 “*must-link*”, update 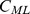 :
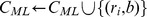
• if 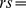 “*cannot-link*”, update 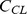 and cluster 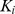 first:
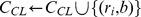 ; 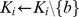
then query the relationships between *b* and other representatives *r_j_* 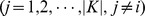 :
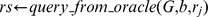
• if 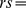 “*cannot-link*”, update 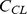 :
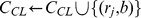
• if 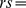 “*must-link*” to certain 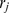 , update 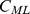 and cluster 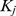 :
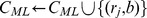 ; 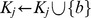
10: Repeat step 9, until certain user-specified criteria are met
11: **return** 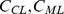

The function 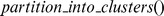 in Algorithm 2 is responsible for partitioning the candidate node set, *cand*, into some clusters. In this function, we take every node in set 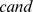 as a cluster first, then merge some clusters repeatedly to obtain the resulting clusters. The logic of this function is described as Algorithm 3 in Table 3.

**Table 3:** Algorithm 3: 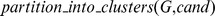.

** Input:** 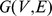 , the network; 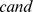 , the set of candidate nodes
** Output:** 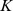 , the set of clusters
1: For each node pair 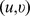 , where 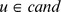 , 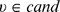 , calculate the value of 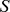 :
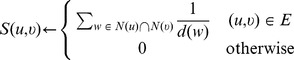
2: For each node 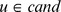 , identify its *most similar neighbors* (abbreviated as *msn*):
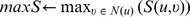
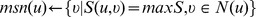
3: Construct the initial clusters 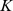 by taking each node 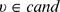 as a cluster:
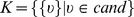
4: Select the node with the largest degree from the unprocessed nodes in 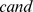 as the 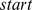 node
5: Merge the cluster containing the 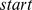 node and every cluster containing any node in 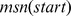
6: Take each node in 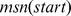 as a new 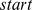 node alternately, and repeat step 5 until every new 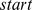 node and 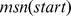 are in the same cluster
7: Repeat steps 4–6 until all of the nodes in 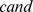 are processed
8: **return** 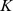

To achieve the goals efficiently, in Algorithm 3, we first calculate a value, *S*, for each pair of nodes 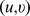 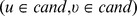 using the following formula:
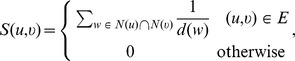
the value of 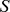 takes the role of the *local similarity* between the pair of nodes in 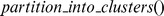. Next, for every node in set *cand*, the *most similar neighbors* are identified according to the value of local similarity 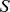. Each node in set 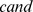 is then taken as a cluster, and that node is the sole member of the corresponding cluster. In the next steps, two clusters are merged into one iteratively, until all nodes in set 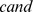 are processed. In each merge operation, the nodes contained in one of the two clusters are some of the *most similar neighbors* of the nodes contained in the other cluster. Finally, the set of clusters is returned and used in Algorithm 2.

### Similarity measure computation algorithm based on random walk

In Algorithm 1, we expand the communities by selecting the most similar unclassified nodes and inserting them into the corresponding community iteratively. In general, the selected unclassified nodes fall into two categories: nodes having *must-link* relationships with the classified nodes, and nodes having the largest similarity values with the corresponding communities among all of the community and unclassified node pairs. Because of the small number of *must-link* constraints, the vast majority of nodes are pulled to join the communities for the latter reason.

Thus, the similarity between a community and a node plays an important role in our algorithm. According to Definition 8, 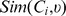 is defined as the maximal similarity value between every node in community 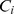 and node 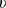, and thus we need to compute the similarity 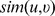 between every pair of nodes, 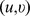, in the network beforehand, where 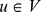 and 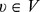.

Adapting Algorithm 1, we need the similarity to provide a quantitative metric to measure the closeness between two nodes from the global perspective of the entire network. When the length of the random walks is set properly, a random walker starting from any node can walk through the whole network, and thus the idea of random walk can be used to compute the global similarity between any pair of nodes. Most of the methods based on random walk implicitly utilize the tendency of the walker being trapped in a group of densely connected nodes corresponding to a community by using the probabilistic theory knowledge and matrix operations. In [35], the authors implemented a method directly applying the idea of random walk by actually simulating the process of random walk in a network to compute the similarities between nodes. In this paper, we directly utilize such method to compute the similarity values used in Algorithm 1, the operations of this random walk method are listed as Algorithm 4 in Table 4.

**Table 4:** Algorithm 4: Similarity computation algorithm based on random walk.

** Input:** 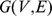 , the network; 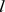 , the length of the random walks
** Output:** 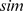 , the similarity matrix whose elements are the similarity values between nodes in the network
1: Initialize the similarity matrix:
** for** 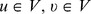 **do**
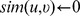
2: Take any node in the network as the *start* node to simulate a random walk, and record the visited nodes during the walk in set 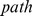 :
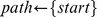
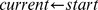
** for** 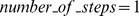 **to** 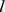 **do**
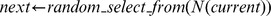
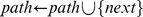
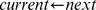
3: Increase the similarity value between every pair of nodes recorded in set 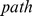
** for** 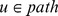 **do**
** for** 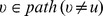 **do**
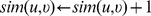
4: Take any other node as the 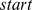 node, repeat the random walk and similarity-increase operations depicted in steps 2 and 3, until every node in the network is taken as the 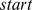 node to simulate a random walk once
5: **return** 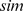

The operations are almost self-explanatory. First, all elements of the similarity matrix 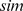 are initialized to be 0. We then take every node in the network as the 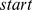 node to carry out a random walk. During each random walk, we keep track of the visited nodes into set 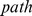, and at the end of each walk, the similarity value between each pair of nodes in 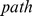 is increased. After all random walks are completed, we finally obtain and return the similarity matrix, 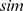.

Clearly, Algorithm 4 applies to undirected networks only, because we need the walker starting from any node can walk through the whole network in principle. In many directed networks, it is impossible. In addition, the networks should be unweighted networks, or the walker have to consider the influences of the edge weights in each jump. Because the edge weights in different networks have different meanings, this will increase the complexity of the similarity computation. For simplicity, Algorithm 4 does not touch upon the edge weights at all. Therefore, Algorithm 4 applies to unweighted networks only also. This is also the major reason why we only consider the undirected and unweighted networks in this paper.

## Evaluation metrics

Although the algorithm can consistently produce a partition of a network, how do we know whether the partition is acceptable as a community structure or not? We need some metrics to measure the quality of the community structure extracted by the algorithm. The *modularity* [7] is the de facto standard at present to measure the strength of a community structure, the *accuracy* and 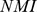 (*Normalized Mutual Information*) [51] are two metrics frequently used to assess the performance of clustering algorithms in the fields of data mining and machine learning. To some extent, the essence of detecting a community structure from a network is node clustering, thus using the *accuracy* and 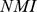 to measure the ability of the community detection algorithms also makes sense. Therefore, in this paper, we take all of the three metrics to evaluate the ability of the algorithms.

## Modularity

As mentioned above, The *modularity*, denoted as 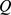, is the actual metric at present to measure the quality of a community structure. Let us assume that a network is partitioned into 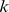 communities, and define a 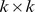 symmetric matrix 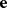, whose element 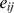 is the proportion of edges in the network that connect the nodes in community 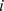 with the nodes in community 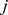. Further, let us define the row sum of 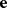 as 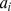, i.e., 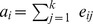, which represents the proportion of edges that are incident to nodes in community 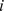. Based on the assumption and definitions, the metric *modularity* is defined as:
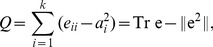
(1)where the first term, 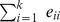, is the proportion of edges inside the communities, and the second term, 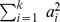, represents the expected value of the same quantity in a random network constructed by keeping the same node set and node degree distribution, but connecting the edges between nodes randomly. Such randomness is generally accepted as a network having no significant community structure.

The *modularity* 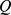 measures the quality of a community structure from the perspective of how far it deviates from a random network: the more the value of 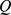 is close to 0, the more the term 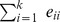 is close to 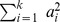, which means that the network more approaches a random network, and thus the strength of the community structure is weaker. In contrast, the larger the value of 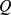 is, the further the community structure deviates from a random network, and thus the strength of the community structure is stronger. In practice, values greater than about 0.3 have already indicated significant community structures, and typically fall within the range of 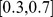. Higher values of 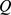 are rare.

The *modularity* can be computed using only the topological connectivity of the network, without requiring any other information. However, some disadvantages of the *modularity* exist: in [52], the authors found that optimizing the *modularity* in large networks would fail to identify communities that are smaller than a scale, even when the smaller communities is well defined. This is the so-called resolution limit problem. Furthermore, the *modularity* formalizes the goal of attaining high intra-community connectivity and low inter-community connectivity, and is an *internal criterion* for measuring the quality of a community structure. Regarding the internal criterion, it is well known that a good score does not necessarily translate into a good effectiveness in practice. For the *modularity*, a high value of 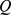 does not necessarily correspond to a real community structure, which will be verified through the experimental results.

Therefore, in addition to the *modularity*, we use the *accuracy* and 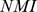 to measure the ability of the community detection algorithms.

### Accuracy

Compared with the *modularity*, the *accuracy*, denoted as *A*, is an *external criterion* for evaluating the ability of the community detection algorithms, and is defined as the ratio of the number of nodes classified into the correct communities to the total number of nodes in the network. As mentioned above, community detection is equivalent to node clustering in the network to some extent, thus it is a basic requirement that the nodes be classified into the correct communities. The *accuracy* takes the ground truth community structure as a baseline, and utilizes the ratio to measure the proximity between the extracted community structure and the ground truth community structure, and to measure the ability of the algorithm.

Let us denote the ground truth community structure and the extracted community structure as 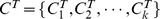 and 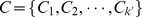, respectively. To compute the *accuracy*, we assign every community 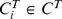 a unique label, which is also assigned to each node 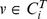 concurrently as its true label, denoted as 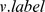. We then resolve which community 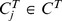 matches with community 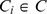. To do so, for each community 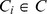, we scan all of the nodes in 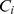 to count the occurrences of each label in 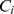, and take the label occurring most frequently in 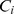 as the label of community 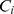. After this process, some communities may have the same labels. For these communities, we keep the community with the largest number of nodes with the same label, and for each of the other communities, if the nodes in the community have no other labels, that community is removed from 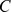, and all nodes in that community are taken as misclassified nodes; otherwise, we take the next label whose node number is the next-largest in the community as the label of that community. If some communities still have the same labels, this procedure is repeated until every community has a unique label. Then, community 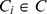 and community 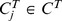 with the same label match with each other, and we assign the label of community 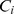 to each node 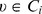 as its predicted label, denoted as 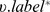. Based on the above description, 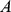 is defined as
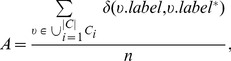
(2)where 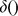 is the Kronecker delta function.

The *accuracy* 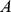 measures how the extracted community structure approaches the ground truth community structure. Obviously, the value of 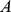 falls within the range of 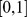, and the more it is close to 1, the more the extracted community structure is close to the ground truth community structure. The ideal scenario is 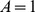, which is corresponding to the result that all nodes in the network are classified into the corresponding communities correctly, so that the extracted community structure is identical to the ground truth community structure.

### NMI

*NMI* is an information-theory based metric, which measures the quality of the extracted community structure from the perspective of the agreement between the extracted community structure and the ground truth community structure, i.e., it also takes the ground truth community structure as a baseline, and thus is also an *external criterion* for measuring the ability of the community detection algorithm.

Taking the frequency counts as approximations of the probabilities, the entropies of the ground truth community structure and the extracted community structure can be represented as 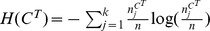 and 
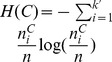
, respectively, where 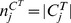, 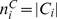. The joint entropy of them can be expressed as 
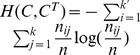
, where 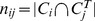, which is the number of shared nodes in 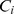 and 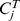. The agreement between the extracted community structure 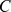 and the ground truth community structure 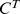 is measured by the mutual information 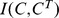, which is defined as follows:
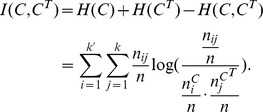



In practice, it is the normalized version of the mutual information that is frequently used to measure the agreement between the extracted community structure and the ground truth community structure, rather than the mutual information itself. It is easy to prove that 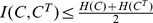, therefore, the normalized mutual information, 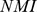, is defined as follows:
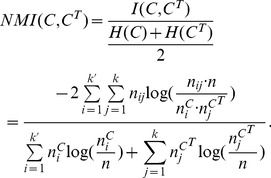
(3)


Clearly, the value of *NMI* also falls within the range of [0, 1], and the larger the value of *NMI* is, the more the extracted community structure agrees with the ground truth community structure, whereas the smaller the value of *NMI* is, the farther they differentiate from each other, and vice versa.

## Results and Discussion

### Datasets

In our experiments, we need to evaluate the results both qualitatively and quantitatively, thus the networks used for the evaluation have to satisfy certain criteria: their ground truth community structures must be known a priori, their scales must be sufficiently small to facilitate the interpretation and visualization of the results, and the networks should be publicly available to facilitate the verification of the methods or algorithms. These restrictions resulted in the selection of four real-world networks, i.e., Zachary's karate club network [6]–[8], [53], Lusseau's bottlenose dolphin social network [54], a map used in the board game Risk [35], and a collaboration network of scientists working at the Santa Fe Institute, which is an interdisciplinary research center in Santa Fe, New Mexico [6]. The statistical information of these networks is listed in Table 5.

**Table 5:** Statistical information of the networks.

network	nodes	edges	communities
karate	34	78	2
dolphin	62	159	4
Risk map	42	83	6
collaboration	118	197	6

Using these networks, we carried out two types of experiments: one for testifying the ability of the semi-supervised community detection algorithm based on the *must-link* and *cannot-link* constraints, and the other for demonstrating the utility of the semi-supervised component-generation algorithm based on active learning.

### Parameter settings

In Algorithm 2, the 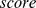 threshold, 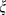, works as a parameter to control the number of nodes extracted into the candidate set, 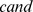. Too large 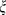 will filter out too many nodes with larger degrees, this will lead to the result that the selected nodes cannot distribute over all of the ground truth communities. On the contrary, too small 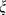 will extract too many nodes into set 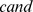, this will influence the efficiency of partitioning set 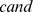 into some clusters. In the following experiments, we controlled the value of 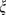, so that the nodes whose 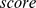 are among the top 50% of 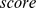 values were extracted into set 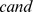, and then 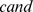 was quickly partitioned into some clusters.

In Algorithm 4, the length of the random walks, *l*, is also a parameter. In our experiments, we accepted the setting, *l* = *n*, as what is used in [35], so that the walker starting from any node can reach any other node in the network, theoretically. Therefore, the similarity between any two nodes in the network can be computed.

### Experiments on the ability of semi-supervised community detection algorithm

To test the ability of our semi-supervised community detection algorithm, we ran the proposed algorithm on the four networks described above, and compared the results with those of four unsupervised community detection algorithms, Fast*Q*, LPA, Infohiermap, and PPC. For our proposal, the initial skeleton of the community structure is constructed from the *must-link* and *cannot-link* constraints, and as the minimum requirement, the nodes that are selected to generate these constraints should distribute over all of the ground truth communities. Thus, to accommodate this minimum requirement, in these experiments, we controlled the termination criteria of the active node selection approach in Algorithm 2, and selected only the nodes with the maximal degrees in the corresponding clusters to query their relationships. As for LPA, it is a non-deterministic algorithm, running the algorithm on a given network many times may incur different results. We therefore took the method originated in [27] to run the LPA 30 times on every network, and then aggregated these community structures to obtain the resulting structure. But to be frank, the aggregated structure on each network is still non-deterministic, and in the experiments described below, we therefore performed the aggregation operations 20 times on every network, and the aggregated community structure occurring most frequently was taken as the resulting structure of that network.

#### Zachary's karate club network

**Figure 2:**
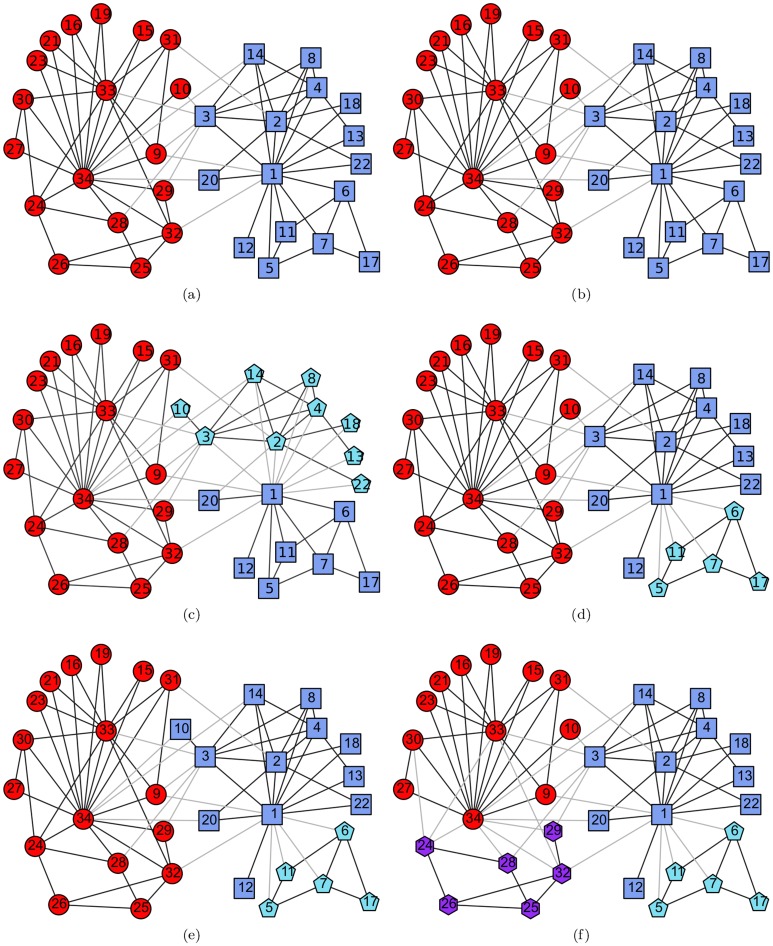
Zachary's karate club network. (a) The ground truth community structure; (b) The community structure extracted by the proposed algorithm; (c) The community structure extracted by Fast*Q*; (d) The community structure aggregated from 30 community structures extracted by LPA; (e) The community structure detected by Infohiermap; (f) The community structure identified by PPC.

This is a well-known benchmark network for testing community detection algorithms. The network is made up of 34 nodes and 78 edges, where every node represents a member of a karate club at an American university. If two members are observed to have social interactions within or away from the karate club, they are connected by an edge. Later, because of a dispute arising between the club's administrator and instructor, the club is eventually split into two factions centered on the administrator and the instructor, respectively. Matched with these two factions, the ground truth community structure is illustrated in Figure 2-(a). Feeding this network into the proposed and comparison algorithms, we obtained the results illustrated in Figures 2-(b), 2-(c), 2-(d), 2-(e), and 2-(f), respectively. The comparison results of the three metrics are listed in Table 6.

**Table 6:** Comparisons of the 3 metrics: A rank (number in parentheses) is attached to the value of each metric for each network, and the value with the highest rank for each metric on each network is shown in bold.

network	algorithm	*Q*	*A*	*NMI*	rank score	rank
karate	ground truth	0.371	1.00	1.00		
	Fast*Q*	0.381 (4)	0.735 (4)	0.692 (4)	4.00	5
	LPA	0.399 (3) (max: 0.416 average: 0.397)	0.853 (2)	0.826 (2)	2.33	**1**
	Infohiermap	0.402 (2)	0.824 (3)	0.699 (3)	2.67	3
	PPC	**0.42** (1)	0.676 (5)	0.687 (5)	3.67	4
	proposal	0.371 (5)	**1.00** (1)	**1.00** (1)	2.33	**1**
dolphin	ground truth	0.519	1.00	1.00		
	Fast*Q*	0.491 (5)	0.839 (4)	0.733 (5)	4.67	5
	LPA	0.503 (4) (max: 0.526 average: 0.506)	0.823 (5)	0.837 (3)	4.00	4
	Infohiermap	0.525 (2)	0.887 (2)	**0.898** (1)	1.67	2
	PPC	0.519 (3)	0.871 (3)	0.812 (4)	3.33	3
	proposal	**0.526** (1)	**0.935** (1)	0.85 (2)	1.33	**1**
Risk map	ground truth	0.621	1.00	1.00		
	Fast*Q*	0.625 (2)	0.929 (2)	0.894 (3)	2.33	3
	LPA	0.624 (3) (max: 0.634 average: 0.619)	0.81 (4)	0.848 (4)	3.67	4
	Infohiermap	**0.634** (1)	0.857 (3)	0.945 (2)	2.00	**1**
	PPC	0.621 (4)	0.81 (4)	0.803 (5)	4.33	5
	proposal	0.621 (4)	**1.00** (1)	**1.00** (1)	2.00	**1**
collaboration	ground truth	0.739	1.00	1.00		
	Fast*Q*	0.749 (2)	0.831 (3)	0.867 (3)	2.67	3
	LPA	0.681 (5) (max: 0.726 average: 0.678)	0.627 (5)	0.799 (5)	5.00	4
	Infohiermap^1*st*^	0.651 (6)	0.636 (4)	0.764 (6)	5.33	6
	Infohiermap^2*nd*^	0.704 (4)	0.602 (6)	0.805 (4)	4.67	5
	PPC	**0.751** (1)	0.847 (2)	0.876 (2)	1.67	**1**
	proposal	0.72 (3)	**0.873** (1)	**0.877** (1)	1.67	**1**

Infohiermap^1*st*^ and Infohiermap^2*nd*^ represent the first-level and the second-level community structures extracted by the Infohiermap algorithm, respectivly.

To obtain the illustrated results, we controlled the termination criteria in Algorithm 2, such that only nodes “1” and “34” were selected to generate the *must-link* and *cannot-link* constraints by accessing the oracle. Clearly, the relationship between this pair of nodes is “*cannot-link*”. Based on this constraint, our method identified the correct community structure from this network easily, the result of which is identical to the ground truth community structure. Compared with this, all of the community structures extracted by Fast*Q*, LPA, Infohiermap, and PPC have some deviations from the ground truth. This means that by introducing only the minimum semi-supervised components, we can obtain the best community structure.

It is worth noting that the output of Fast*Q* herein is different from the counterpart described in [8]. In [8], when the value of modularity *Q* reaches its peak (*Q* = 0.381), the dendrogram agglomerated by the algorithm is cut into two communities correspondingly. However, in our experiments, we carried out the algorithm using a variety of implementations, including conducting the programming ourselves, compiling the source code downloaded from a Web site [55], running the executable file, and calling the function implemented in **igraph** package [56]. All outputs are consistent with that presented herein, i.e., when *Q* = 0.381, the corresponding structure contains three communities, as illustrated in Figure 2-(c), rather than two.

In Table 6, all of the values of *Q* obtained by Fast*Q*, Infohiermap, PPC, and the maximal, the average and the aggregated values of *Q* acquired by LPA are larger than that of the ground truth community structure, but all of the corresponding community structures deviate from the ground truth community structure more or less, which confirms one of the shortcomings of the modularity mentioned before.

#### Lusseau's bottlenose dolphin social network

**Figure 3:**
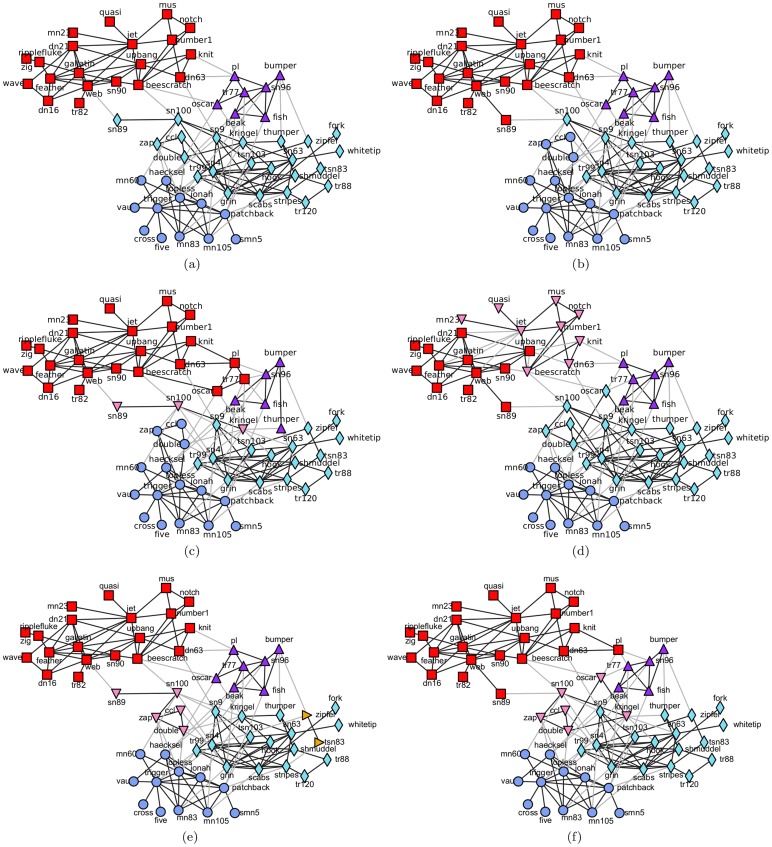
Lusseau's bottlenose dolphin social network. (a) The ground truth community structure; (b) The community structure extracted by the proposed algorithm; (c) The community structure identified by Fast*Q*; (d) The community structure aggregated from 30 outputs of LPA; (e) The community structure detected by Infohiermap; (f) The community structure identified by PPC.

This is also a famous network widely used as a benchmark to validate community detection algorithms. It contains 62 nodes that represent bottlenose dolphins living in Doubtful Sound, New Zealand, and 159 edges that represent associations between dolphin pairs observed to co-occur more often than expected occasionally. The nodes in this network can be partitioned into four groups, which corresponds to the ground truth community structures illustrated in Figure 3-(a). Running our proposed algorithm and the comparison algorithms on this network, we obtained the results illustrated in Figures 3-(b), 3-(c), 3-(d), 3-(e), and 3-(f). The comparison results of the three metrics are listed in Table 6.

In this network, the nodes with the maximal degrees in the clusters selected by Algorithm 2 are nodes “grin”, “topless”, “web”, “jet”, “tr77”, and “double”. Among them, nodes “grin” and “double” belong in the same ground truth community, as do the pair of nodes “web” and “jet”. To meet the minimum requirement that the selected nodes simply cover all of the ground truth communities, we interfered manually to select the node whose degree is larger than the other node in the pair. When the two nodes had the same degree value, the one with the larger 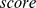 value was selected. Thus, the semi-supervised components were generated from nodes “grin”, “topless”, “web”, and “tr77” in this experiment, and it is clear that the relationships between their pairs are all “*cannot-link*”s. Compared with the ground truth community structure shown in Figure 3-(a), in the result of our proposed method illustrated in Figure 3-(b), nodes “sn89”, “zap”, “double”, and “ccl” were classified into the wrong communities. The first 3 of them are all located at the community boundaries, and they tend to be classified erroneously. For the misclassifications of nodes “double” and “zap”, node “ccl” also becomes a boundary node. Thus, it is easy to understand why they were classified into the wrong communities.

Despite this, from the perspective of the proximity of the community structures identified by the algorithms and the ground truth community structure, the proposed algorithm performs better than the other algorithms. Both the values of *Q* and *A* are larger in the proposed algorithm than in the comparison algorithms, and the value of *NMI* of the proposed algorithm is only smaller than that of the Infohiermap algorithm, but still larger than those of the others. Additionally, along with the increase in the number of selected nodes participating in generating the semi-supervised components, some of the misclassifications will be eliminated, the value of *Q* will approach that of the ground truth community structure, the values of *A* and *NMI* will increase further, all of which are verified in the next type of experiments.

#### Risk map network

**Figure 4:**
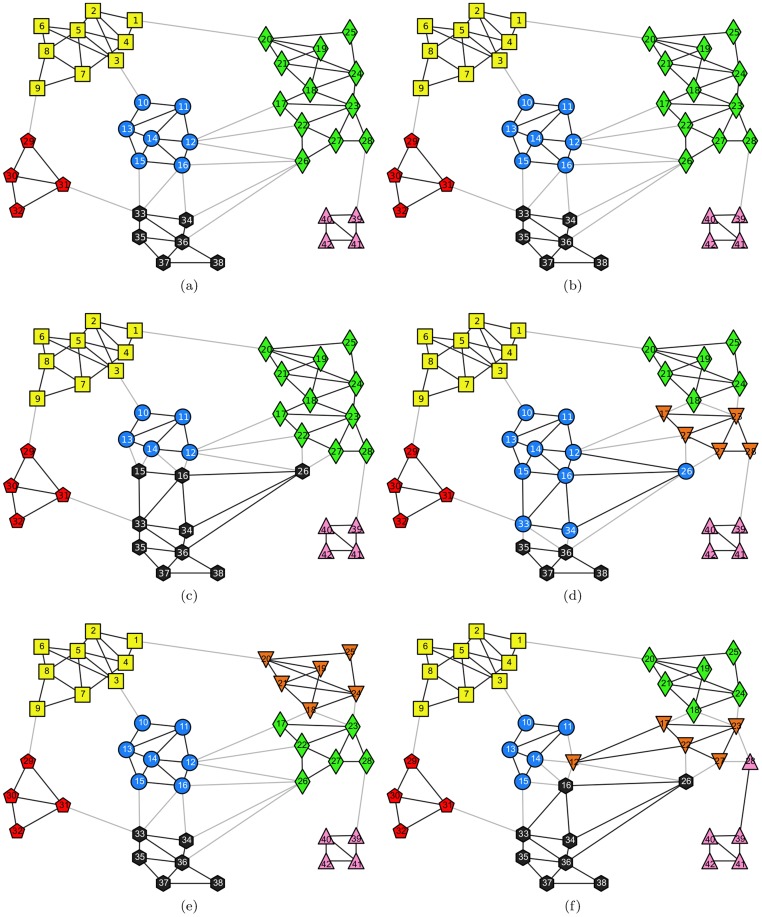
Risk map network. (a) The ground truth community structure; (b) The community structure identified by the proposed algorithm; (c) The community structure extracted by Fast*Q*; (d) The community structure aggregated from 30 outputs of LPA; (e) The community structure detected by Infohiermap; (f) The community structure extracted by PPC.

This network is a map of the popular board game Risk, which was invented by Albert Lamorisse and released in 1957 originally. The game can be played by two to six players on a board representing a political map of the Earth, which is divided into 42 territories grouped into 6 continents. Hence, this network is composed of 42 nodes and 83 edges, and all nodes can be partitioned naturally into 6 communities. To eliminate any political sensitivity, we assigned each of the nodes a continuous number instead of the name of the country or territory, the ground truth community structure of which is shown in Figure 4-(a). Taking this network as an input in the proposed and comparison algorithms, the outputs are demonstrated in Figures 4-(b), 4-(c), 4-(d), 4-(e), and 4-(f), individually, and the comparison results of the three metrics are enumerated in Table 6.

In this network, the minimum number of nodes with the maximal degrees in the clusters selected by Algorithm 2 are nodes “5”, “36”, “24”, “31”, “40”, and “16”, and all relationships between every pair of them are *“cannot-link”*s. Based on these constraints, the proposed algorithm yielded the result shown in Figure 4-(b), which is identical to the ground truth community structure. This means that, by utilizing the minimum number of semi-supervised components to guide the community detection procedure, we obtain the best result for this network.

The 6 communities in this network are well separated, but because of the existence of some special nodes, some mistakes tended to be introduced into the results of many algorithms. For instance, node “26” is such a special node, which has 6 edges, but only 2 of them are intra-community connections. For 4 other inter-community edges, 2 of them connect nodes in another community, and 2 other of them are incident to nodes in the third community. Thus, it is hard to say which one of the three communities the node is more intimate with. Similar scenarios occur for nodes “12”, “16”, and “33”. It seems rational that they be classified into any one of the communities that they are associated with, if we do not consider the physical meaning of the nodes in this network. The results produced by the comparison algorithms have certain biases from the ground truth community structure, and most of the mistakes occur around these nodes.

For our proposed method, a special node, “16”, was selected to participate in the generation of the semi-supervised components. Because the relationship between nodes “16” and “36” is “*cannot-link*”, as is the relationship between nodes “16” and “24”, and the similarity values computed by Algorithm 3 indicated that nodes “33” and “34” were more intimate with node “36” than with node “16”, and that node “26” was closer to node “24” than to node “16” or node “36”, and thus the misclassifications of these nodes were eliminated. The resulting structure identified by our proposed method is already identical to the ground truth community structure, and naturally, the values of the three metrics of our algorithm are superior to those of the comparison algorithms. In fact, if more semi-supervised components are needed, nodes “23”, “26”, “12”, “33”, “18”, *etc.* will be selected by Algorithim 2 individually to generate the semi-supervised components.

#### Scientist collaboration network

**Figure 5:**
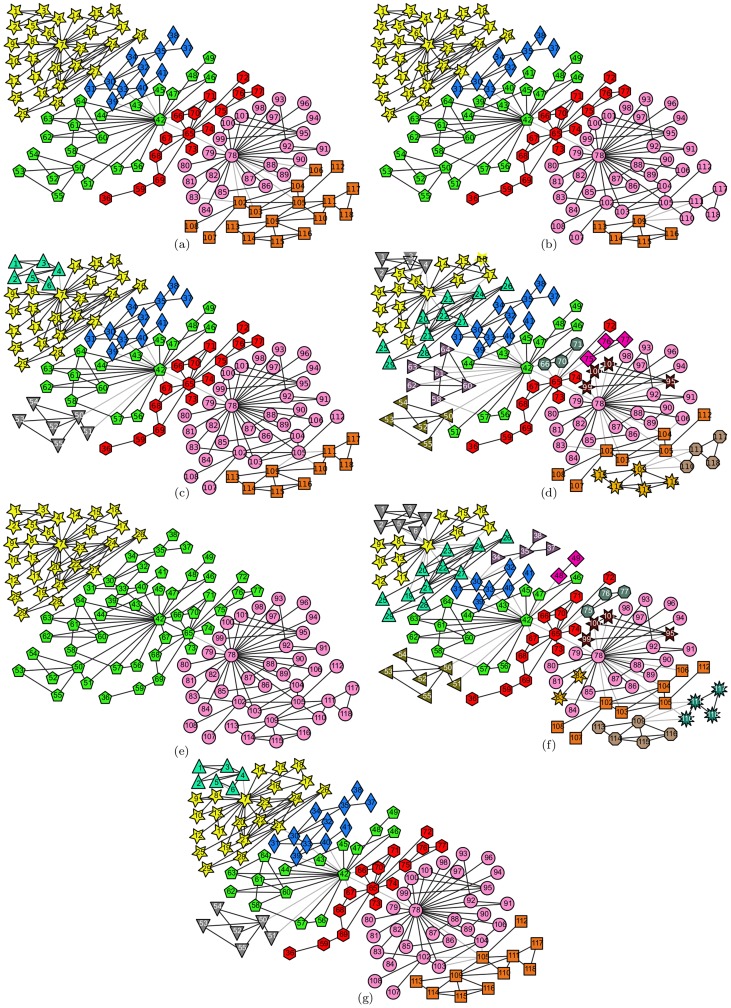
Collaboration network of scientists at the Santa Fe Institute. (a) The ground truth community structure; (b) The community structure detected by the proposed algorithm; (c) The community structure obtained by Fast*Q*; (d) The community structure aggregated from 30 results of LPA; (e) The first-level community structure extracted by Infohiermap; (f) The second-level community structure extracted by Infohiermap; (g) The community structure identified by PPC.

This network is the largest component of a collaboration network of scientists in residence at Santa Fe Institute. Here, the nodes represent the scientists, and the edges connect those scientists who have coauthored at least one article. This network contains 118 nodes and 197 edges, and can be divided into 6 partitions as its ground truth community structure, which is as presented in Figure 5-(a). Feeding this network into the algorithms, we achieved the final results visualized in Figures 5-(b), 5-(c), 5-(d), 5-(e), 5-(f), and 5-(g), separately. The comparison results of the three evaluation metrics are listed in Table 6.

In this network, nodes “78”, “42”, “7”, “65”, “109”, “33”, “111”, and “75” were chosen by Algorithm 2. In the ground truth community structure, nodes “75” and “65” belong to the same community, and as do nodes “109” and “111”. To meet the minimum requirement that the selected nodes simply cover all of the ground truth communities, we also manually interfered and selected from the two node pairs the node with the larger degree, i.e., in this experiment, nodes “78”, “42”, “7”, “65”, “109”, and “33” were selected to generate the semi-supervised components. Apparently, all of the relationships between each pair of nodes are “*cannot-link*”s. Utilizing these constraints, the proposed algorithm extracted the community structure shown in Figure 5-(b).

Compared with the ground truth community structure shown in Figure 5-(a), 15 nodes (“39”, “40”, “41”, “102”, “103”, “104”, “105”, “106”, “107”, “108”, “110”, “111”, “112”, “117”, and “118”) were classified into incorrect communities. Some of them, including nodes “39”, “40”, “102”, “103”, “104”, and “105”, are located at the community boundaries. Among the neighbors of each of them, there exists a node with a very large influence who acts like a center of gravitation. For the first two boundary nodes, node “42” plays this role; and for the latter four nodes, node “78” is the authority. In the random walks passing through those boundary nodes, the walker is more likely to be attracted by these two centers to depart from the communities where the boundary nodes originally belonged, and to be trapped in opposite communities, thus these boundary nodes tend to be misclassified into the opposite communities. Owing to the mistakes this introduces, misclassifications of the other nodes (“41”, “107”, “108”, “106”, “110”, “111”, “117”, and “118”) are inevitable. Along with the increase in the number of selected nodes, most of these boundary nodes will be taken as the nodes with least certainty and be selected to generate the *must-link* and *cannot-link* constraints, thus the vast majority of their misclassifications will be eliminated, which is verified by the next type of experiments we conducted.

Although, these nodes were misclassified by the proposed algorithm, the resulting structure of the proposed algorithm is the closest to the ground truth community structure compared with the other algorithms. Fast*Q* took apart two small groups of nodes from two larger communities, and took them as two additional communities; in addition, 8 other nodes (“108”, “107”, “102”, “103”, “105”, “104”, “106”, and “112”) were also misclassified into the incorrect communities. For LPA, its resulting structure is quite poor, in addition to some nodes being assigned to the incorrect communities, many small groups of nodes were separated from the larger communities, and the resulting structure deviates far from the ground truth community structure. Infohiermap extracted two levels of community structures from this network, the first level contains 3 communities, which is shown in Figure 5-(e), and the second level consists of 16 communities, which is illustrated in Figure 5-(f). Both of them depart far from the ground truth community structure. For PPC, the resulting structure is somewhat similar with that of Fast*Q*, except for the community assignments of nodes “105”, and “112”. Therefore, it is still not an ideal result.

All values of the three evaluation metrics of the community structures extracted by the proposed algorithm and the comparison algorithms are listed in Table 6. Here, PPC obtained the largest *modularity* twice (on the karate club network and scientist collaboration network), both Infohiermap and the proposed algorithm obtained the largest *modularity* once (on the Risk map network and on the dolphin social network, respectively). However, as discussed above, all of the community structures corresponding to these largest modularities have certain deviations from the ground truth community structures, which verifies one of the previously mentioned shortcomings of the *modularity*. However, the proposed algorithm achieved the largest *accuracy* on all four networks, got the largest *NMI* on three networks and the second largest *NMI* on the other network. When considering the meanings of the *accuracy* and *NMI*, this result indicates that the community structure extracted by the proposed algorithm is the closest to the ground truth community structure, i.e., by introducing only the minimum semi-supervised components, we can obtain the best results. This indicates the effectiveness and significant ability of our proposed semi-supervised community detection algorithm. For another perspective, we attached a rank (the number in the parentheses) to each of the metrics of each network, calculated a score by averaging these ranks for every algorithm, and used the score to rank the algorithms. From the ranks listed in the last column of Table 6, we can confirm that the proposed semi-supervised algorithm is superior to the comparison algorithms in its ability to detect the community structures from networks.

### Experiments on the utility of the semi-supervised component generation strategy

In this subsection, we demonstrate the utility of our proposed semi-supervised component generation strategy based on active learning. In Algorithm 2, we loosened the termination criteria step by step, thus the number of selected nodes and then the number of generated semi-supervised components increased gradually. Each time the semi-supervised components were generated, we integrated them in Algorithm 1 as constraints to guide the community detection process. Meanwhile, we applied a random-selection strategy to select an equal number of nodes to generate the semi-supervised components, and then incorporated them in Algorithm 1 to detect the community structure from the network as a comparison. Here, two kinds of random-selection strategies were employed: selecting the nodes from the network completely at random (denoted as “random 1”), and selecting the nodes randomly but ensuring that the selected nodes cover all of the ground truth communities (denoted as “random 2”). When the community structures were extracted from each network, we applied comparisons using *Q*, *A*, and *NMI* to determine which strategy can produce the result closest to the ground truth community structure. In this way, we demonstrated that the proposed semi-supervised component generation strategy based on active learning can actively acquire the *must-link* and *cannot-link* constraints with the maximum utility for the proposed community detection algorithm, thus showing that our proposal is an effective method for extracting high-quality community structures from networks.

As described in the first type of experiments, incorporating only the minimum number of semi-supervised components, the community structures detected from the karate club network and the Risk map network by the proposed method are identical to the ground truth community structures. This means that the experiments effectively demonstrated the utility of the proposed active semi-supervised component generation strategy on these networks, and it was unnecessary to further increase the number of selected nodes. Thus, we conducted the following experiments only on the dolphin social network and the scientist collaboration network.

There are 4 and 6 communities in the ground truth community structures of these two networks, respectively, but as described in the previous subsection, the minimum numbers of nodes selected from these networks by Algorithm 2 were 6 and 8, respectively. In the first type of experiments, to accommodate the minimum requirement that the selected nodes distribute simply over all of the ground truth communities, we interfered manually to choose 4 of the selected nodes from the dolphin social network, and 6 of the selected nodes from the scientist collaboration network, to generate the semi-supervised components.

**Figure 6:**
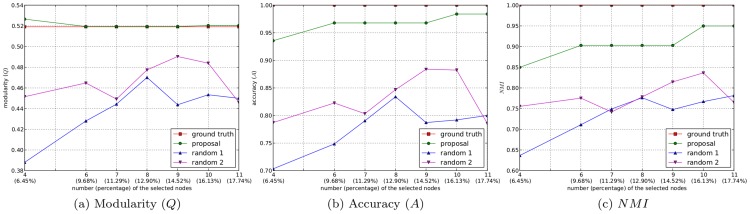
The evolutions of the three metrics on the dolphin social network.

**Figure 7:**
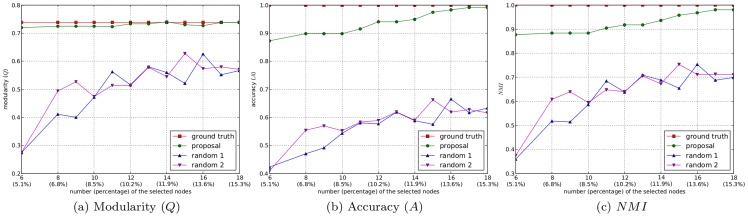
The evolutions of the three metrics on the scientist collaboration network.

However, no minimum limit is needed in this type of experiments, and thus we loosened the termination criteria in Algorithm 2 step by step, such that the number of nodes selected to generate the semi-supervised components increased one by one. Each time the semi-supervised components were generated, we incorporated them in Algorithm 1 to obtain the resulting community structure. This process ended when the values of *A* and *NMI* no longer increased. For the two random methods, the selected nodes were non-deterministic. To eliminate the occasionality, we repeated the two random methods 10 times each for each number of selected nodes, and took the average values of *Q*, *A*, and *NMI* as the resulting values of the three metrics. In this way, for dolphin social network, we carried out 6 groups of experiments starting from 6 selected nodes, and increased one selected node each time. The evolutions of the values of *Q*, *A*, and *NMI* corresponding to the community structures extracted by the proposed method and the two random methods are shown in Figure 6. For the scientist collaboration network, starting from 8 selected nodes, we carried out 11 groups of experiments by adding one node into the selected node set each time. The evolutions of the values of *Q*, *A*, and *NMI* of the extracted community structures from this network are illustrated in Figure 7. To maintain the completeness of the experiments, the values of the three metrics corresponding to the scenarios that the minimum number limit is met are also plotted in Figures 6 and 7.

In both Figures 6 and 7, all values of *Q*, *A*, and *NMI* of the community structures extracted by the proposed algorithm are significantly larger than the counterparts of the two random methods. For the proposed algorithm, along with the increase in the number of selected nodes, the values of *Q* approach those of the ground truth community structures, and the values of *A* and *NMI* increase steadily. When the number of selected nodes is increased to 10 in the dolphin social network (about 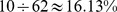 of the total nodes in the network) and to 17 in the scientist collaboration network (about 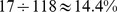 of the total nodes in the network), the values of *A* and *NMI* reach their peaks, and the extracted community structures are almost identical with the ground truth community structures (only 1 node was misclassified in both of the two networks). However, for the two random methods, the values of all three evaluation metrics fluctuate along with the increase in the number of the selected nodes, and even when *A* and *NMI* get their peak values, more than 12% of the nodes in the networks still cannot be assigned to the correct communities. These comparisons show that the proposed active learning algorithm can generate the semi-supervised components with the maximum utility from the networks.

## Conclusions

In this paper, we introduced active learning into the problem of community detection, and presented a community detection method, which is a combination of a semi-supervised community detection algorithm and a *must-link* and *cannot-link* constraint generation strategy based on active learning. In the semi-supervised community detection algorithm, the skeleton of the initial community structure is constructed from the nodes involved in the *must-link* and *cannot-link* constraints first. The (*community, unclassified node*) pair with the largest similarity value is then identified, and that *unclassified node* and all of its *must-link* and transitive *must-link* partners are inserted into the *community* repeatedly, until all nodes in the network are assigned to the corresponding community. In this way, we obtain the final community structure. To acquire the high-quality *must-link* and *cannot-link* constraints, a semi-supervised component generation algorithm was proposed. We first calculate a *score* value for every node in the network, and the nodes whose *score* values are larger than a given threshold, 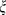, are then extracted into a node set from the network. Next, this node set is quickly partitioned into some clusters, and the nodes with the maximal degrees in each cluster, along with the boundary nodes of each cluster, are selected step by step, and the *must-link* and *cannot-link* constraints are finally generated by accessing a noiseless oracle. We also performed extensive experiments on 4 real-world networks, the experimental results illustrate the effectiveness and significant ability of our proposed method.
